# B vitamins related to homocysteine metabolism in adults celiac disease patients: a cross-sectional study

**DOI:** 10.1186/s12937-015-0099-8

**Published:** 2015-10-20

**Authors:** Flávia Xavier Valente, Tatiana do Nascimento Campos, Luís Fernando de Sousa Moraes, Helen Hermana Miranda Hermsdorff, Leandro de Morais Cardoso, Helena Maria Pinheiro-Sant’Ana, Flávio Augusto Barros Gilberti, Maria do Carmo Gouveia Peluzio

**Affiliations:** 1 Departamento de Nutrição e Saúde, Universidade Federal de Viçosa, Av. PH. Rolfs, Campus Universitário, Viçosa, Minas Gerais CEP 36570-900 Brazil; 2 Departamento de Nutrição, Universidade Federal de Juiz de Fora, Campus Governador Valadares, Av. Dr. Raimundo Monteiros Rezende, 330, Governador Valadares, Minas Gerais 35010-177 Brazil

**Keywords:** Celiac disease, Gluten-free diet, Folate, Homocysteine

## Abstract

**Background:**

The only treatment for celiac disease is the gluten-free diet. Few studies have assessed the nutritional adequacy of this diet, especially of B vitamins related to homocysteine metabolism. The aim of this study was to assess the nutritional status and serum concentrations of B vitamins involved in homocysteine metabolism, and to determine whether the dietary intake of these vitamins are meeting Dietary Reference Intakes in celiac patients.

**Methods:**

A cross-sectional study enrolled a total of 20 celiac patients (36.3 ± 13.7 years old; 65 % women), following strict gluten-free diet (GFD) and 39 healthy controls matched by sex and age. The dietary intake was assessed by 3-day food records, and serum concentrations of homocysteine and vitamins B_6_, B_12_, and folate were determined after overnight fasting. Comparisons between the two groups were performed by Student’s *t* test or Mann–Whitney *U*-test, for continuous variables. Pearson’s chi-square test or Fisher’s exact test was used for categorical variables. An alpha level of 5 % were considered significant.

**Results:**

Celiac patients had lower serum folate concentrations (7.7 ± 3.5 ng/mL, *P* < 0.05) than controls. All celiac patients had folate intake below the Estimated Average Requirement (EAR) (130.8 ± 53.6 μg/d). However, only a small proportion of celiac patients had hyperhomocysteinemia.

**Conclusions:**

Celiac patients treated with GFD presented inadequacy of dietary folate intake and low-serum concentrations of folate, suggesting that more attention should be given to the quality of the nutrients offered by the GFD, as it constitutes a lifelong treatment.

## Background

Celiac disease (CD) is a systemic autoimmune disorder developed in genetically predisposed individuals by the ingestion of gluten [1]. CD is characterized by an inflammatory process that occurs in the small intestine resulting in flattening of the villi and loss of absorptive function [2]. The only treatment for CD is the total exclusion of gluten from the diet avoiding its food source, such as wheat, rye, and barley [3].

The majority of celiac patients respond well to the gluten-free diet (GFD). However, there are evidences of nutritional deficiencies in these patients, mainly related to vitamin B complex [4–6].

Folate and vitamins B_6_ and B_12_ are, respectively, substrate and essential cofactors for enzymes in homocysteine metabolism, which is an intermediate metabolite of methionine synthesis pathway [7]. Inadequate intake of those vitamins is the most common cause of high concentrations of serum homocysteine [8]. About two thirds of hyperhomocysteinemia cases are related to low or moderate serum concentration of these vitamins, especially folate [9].

In turn, hyperhomocysteinemia in untreated celiac patients has been linked to common characteristics of this disease such as abortions [10], osteoporosis [11], and cardiovascular disease [12]. Although some authors have shown hyperhomocysteinemia in treated celiac patients [13, 14], few studies have evaluated the role of GFD in providing adequate amounts of nutrients. Thus, the aim of this study was to assess the nutritional status and serum concentrations of B vitamins involved in homocysteine metabolism, and to determine whether the dietary intake of these vitamins is meeting Dietary Reference Intakes (DRI) in treated celiac patients.

## Methods

### Study population

**Fig. 1:**
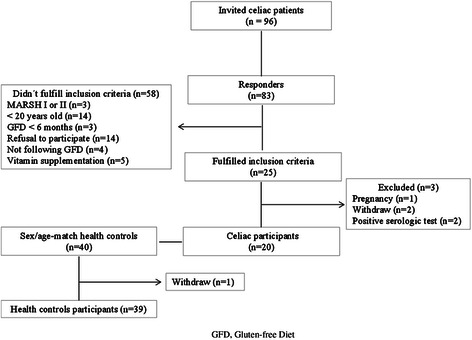
Flow diagram of enrolled participants. GFD, Gluten-free Diet

A cross-sectional, single blind study was carried out with celiac patients recruited from the Nutritional Department of the Health Division at the Universidade Federal de Viçosa between October 2011 and July 2012. All subjects had confirmed diagnosis by duodenal biopsy showing subtotal or total villus atrophy (Marsh III B or C) [15, 16] and were following strict GFD for at least 6 months (Fig. 1). Also, healthy control subjects matched by sex and age with celiac patients were recruited.

In both groups, subjects were excluded if they tested positive for the IgA anti-transglutaminase antibody (≥7.0 U/mL), presented recurrent gastrointestinal symptoms, used vitamins and minerals supplements, as well as pregnant and lactating women. Both groups were studied at the same period, and all measurements were performed at the same clinical chemistry laboratory by the same researcher.

The study was approved by the Ethics Committee on Human Research of the Universidade Federal de Viçosa (Protocol number 146/2011). Written informed consent was obtained from all of the subjects.

### Body composition

Weight and height were measured to calculate the body mass index (BMI) [17]. Subjects with BMI below 24.9 kg/m^2^ were considered eutrophics. The waist circumference was measured at the midpoint between the bottom margin of the last palpable rib and the iliac crest [18]. Total body fat was measured by dual-energy X-ray radiological absorptiometry (DXA, GE Lunar Prodigy, General Electric Medical Systems, Milwaukee, WI, USA). The Fat Mass Index (FMI), calculated by the ration between the weight of fat (kg) and the square of height (m^2^), was used as indicative of body fat adequacy. Results above 6 kg/m^2^ for men and 9 kg/m^2^ for women were considered excess of body fat [19].

### Dietary assessment

Dietary intake was assessed using a three non-consecutive day food records. All volunteers were trained prior to filling out the questionnaire. Subjects were also instructed not to change their usual eating pattern. All records were reviewed by an trained nutritionist. The average daily nutrient intake was calculated using Avanutri Revolution® software version 4.0 (Avanutri & Nutrição Serviços e Informática Ltda., Brazil), using food composition data from TACO [20] and IBGE [21] tables.

For the assessment of folate and vitamin B_6_ intake adequacy, there were considered adequate intake values above the Estimated Average Requirement (EAR) (folate = 320 μg/day; B6 = 1.4 mg/day) and below the Tolerable Upper Intake (UL) (folate = 1000 μg/day; vitamin B6 = 100 mg/day) [22]. As vitamin B_12_ has no UL established, intake above the EAR (2.0 μg/day) was considered appropriate.

### Biochemical assessment

After overnight fast, 20 mL of blood was collected from all the volunteers. The IgA anti-transglutaminase antibody was analyzed by Enzyme- Linked Immuno Sorbent Assay (ELISA) (Celikey® IgA, Phadia AB, Uppsala, Sweden), and homocysteine (Immulite2000, Siemens, USA), vitamin B12, and serum folate (Modular e170, Roche, Switzerland) by chemiluminescence. Vitamin B6 was determined through the analysis of pyridoxal-5-phosphate (PLP), isomer of highest concentration in human plasma, through High-Performance Liquid Chromatography with fluorescence detection [23, 24].

### Statistical analysis

Statistical analyses were performed using Stata software (StataCorp LP, USA, version 9.0). Comparisons between the two groups were performed by Student’s *t* test or Mann–Whitney *U*-test test. The tests depended on the distribution of the variables according to Shapiro–Wilk normality test. For the comparison of proportions, Pearson’s chi-square test or Fisher’s exact test was used. Results were expressed as mean ± standard deviation (s.d.). Values of *P* < 0.05 were considered significant.

The power of the present study was 98 % calculated according to Martínez-González et al. [25] and based on serum concentration of folate as main variable.

## Results

The study comprised of 20 celiac patients and 39 healthy controls, and 65 % were women. Both groups presented eutrophic individuals, according to BMI, but all subjects had excessive body fat, according to FMI (Table 1). The average time for diagnosis and treatment with GFD for celiac patients was 1.2 ± 0.6 years with the mean age at diagnosis of 35.3 ± 13.5 years old. None of the participants reported thyroid pathology, autoimmune disease (exception for celiac patients) or cardiovascular disease.

**Table 1:** Characteristics of celiac patients and healthy controls

Characteristics	Celiac	Control
Participants (*n*)	20	39
Males/Females	7/13	14/25
Age (years)	36.3 ± 13.7	36.0 ± 13.0
BMI (kg/m^2^)	22.5 ± 3.2	23.8 ± 3.7
FMI (kg/m^2^)	10.4 ± 3.1	10.7 ± 3.3
Waist circumference (cm)	76.5 ± 10.3	80.0 ± 11.3
Blood pressure		
Systolic (mmHg)	113.6 ± 2.2	112.9 ± 2.0
Diastolic (mmHg)	74.3 ± 1.9	74.0 ± 1.5
Glycaemia (mmol/L)	4.8 ± 0.1	4.5 ± 0.1
Total Cholesterol (mmol/L)	4.9 ± 0,2	4,6 ± 0.4
HDL cholesterol (mmol/L)	2.7 ± 0.2	2.6 ± 0.1
LDL cholesterol (mmol/L)	6.7 ± 0.2	6.0 ± 0.1
Triglycerides (mmol/L)	1.5 ± 0.2	1.2 ± 0.1

Data are mean ± s.d. *BMI* body mass index, *FMI* fat mass index, *HDL* high density lipoprotein, *LDL* low density liporpotein.*P<  0.05, *t* test or Mann–Whitney *U*-test as appropriate

Celiac patients exhibited lower serum folate concentrations when compared to control group (Table 2), both in men (5.7 ± 1.4 vs 11.7 ± 3.7 ng/mL, *P* = 0.003) and in women (8.8 ± 3.8 vs 13.4 ± 4.3 ng/mL, *P* = 0.002).

**Table 2:** Biochemistry parameters of celiac patients and healthy controls

	Celiac	Control
Participants (*n*)	20	39
Hemoglobin (g/dL)	13.6 ± 1.2	13.4 ± 1.3
Homocysteine (μmol/L)	10.0 ± 3.2	9.3 ± 2.5
Folate (nmol/L)	17.5 ± 8.0*	29.0 ± 9.4
Vitamin B_6_ (nmol/L)	130.3 ± 35.8	130.3 ± 52.3
Vitamin B_12_ (pg/mL)	271.1 ± 89.0	257.3 ± 90.6

Data are mean ± s.d. *P < 0.05 *t* test or Mann–Whitney *U*-test as appropriate

The analysis of dietary intake showed that 51.4 ± 6.9 % of the daily caloric intake of GFD were derived from carbohydrates, 16.2 ± 3.6 % from protein, and 32.1 ± 6.4 % from lipids. The daily caloric intake in the control group was composed of 51.4 ± 9.1 % from carbohydrate, 4.0 ± 16.1 % from protein, and 29.5 ± 5.8 % from lipids, without statistical difference between groups (*P* > 0.05). There were no differences between the groups according to consumption of fiber and vitamins involved in the metabolism of homocysteine either (Table 3).

**Table 3:** Daily intake of energy, macronutrients, fiber, and vitamins of celiac patients and healthy controls

	Celiac	Control
Participants (n)	18	36
Calories (kJ/d)	8460.3 ± 2,559.8	9535.0 ± 4,225.1
Carbohydrate (g/d)	256.8 ± 74.2	273.8 ± 67.3
Protein (g/d)	81.6 ± 28.6	86.6 ± 25.6
Fat (g/d)	74.3 ± 32.1	72.6 ± 23.8
Fiber (g/d)	18.4 ± 7.1	17.8 ± 6.5
Folate (μg/d)	133. 2 ± 62.5	140.9 ± 65.8
Vitamin B_6_ (mg/d)	2.4 ± 1.6	2.6 ± 1.3
Vitamin B_12_ (μg/d)	2.1 ± 1.9	2.4 ± 5.6

Data are mean ± s.d.*P<  0.05), *t* test or Mann–Whitney *U*-test test as appropriate

**Fig. 2:**
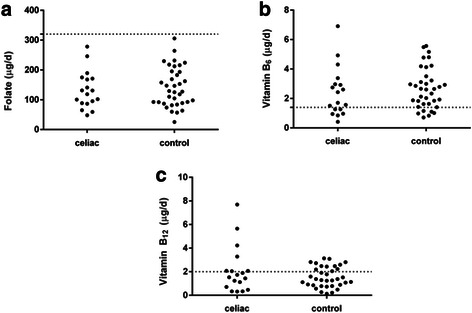
Intake adequacy of folate, vitamin B_6_ and vitamin B_12_ of celiac patients and health controls. **a** folate, **b** vitamin B_6_ and **c** vitamin B_12_. The dotted line (^...........^) indicates the Estimated Average Requirement (EAR) value adopted as reference (folate = 320 μg/day; B_6_ = 1.4 mg/day and B_12_ = 2.0 μg/day). Dots below the dotted line indicates subjects with inadequate intake of each nutrient

However, in both groups 100 % individuals presented inadequate folate consumption (Fig. 2). In the celiac patients, 33.3 (*n* = 6) and 61.1 % (*n* = 11) showed inadequate consumption of vitamins B_6_ and B_12_ compared with 16.6 (*n* = 6) and 64.8 % (*n* = 24) in controls, respectively (*P* > 0.05) (Fig. 2). Also, only 40 % (*n* = 8) of the celiac patients reported conducting nutritional accompaniment.

Despite the inadequate intake and lower serum concentrations of folate, serum homocysteine in celiac patients ranged from 5.3 to 16.3 μmol/L and in controls from 5.2 to 15.3 μmol/L (Table 2). Only 1 patient in each group presented hyperhomocysteinemia (>15 μmol/L). For males, the celiac patients showed an average homocysteine concentration of 11.4 ± 3.13 μmol/L, while the average in the control group it was of 10.7 ± 3.08 μmol/L (*P* > 0.05). For celiac and control females, these values were 9.3 ± 3.12 and 8.5 ± 1.6 μmol/L (*P* > 0.05), respectively.

## Discussion

The results of our study showed that celiac patients presented lower serum concentrations and inadequate folate consumption. However, they did not present hyperhomocysteinemia. Folate deficiency in newly diagnosed and untreated celiac patients is already well described. Dickey et al. [26] observed that newly diagnosed celiac patients presented lower concentrations of eritrocitary and serum folate than controls and celiac patients under GFD for at least 1 year. Wierdsma et al. [27] observed 20 % folate deficiency in these patients, while Saibeni et al. [28] observed 43.5 %. Therefore, celiac patients present risk of developing this deficiency 5.1 times higher than healthy individuals. This is probably related to the loss of proximal small intestine villi resulting in malabsorption of micronutrients in untreated patients. Thus, the higher the degree of vilositary atrophy, the higher the folate deficiency [29].

Adherence to a strict GFD throughout life is the only known treatment for celiac disease [3]. Some factors, such low age at diagnosis, life stages (childhood and adolescence) [30] and higher cost of this diet [31] contribute to treatment non-adherence. Although celiac disease has being a female-predominant disease [32], gender is not a factor that affects the treatment adherence [30, 31] Thus, our population profile was compatible with a good gluten-free diet adherence.

This treatment aims the regeneration of the intestinal villi with reduced risk of complications related to nutrients malabsorption and improvement of nutritional status [33]. The expected histological recovery of the intestinal mucosa is within 6 to 12 months after the onset of GFD [34] resulting in improvement of symptoms [33].

Although some studies have demonstrated improvement on folate status after 6 months [28] and 1 year [35] of treatment, our study showed that even after an average of 1.2 ± 0.6 years of treatment, celiac disease patients still have lower serum folate concentrations when compared to controls. In general, the full recovery of the intestinal mucosa after exclusion of gluten from diet occurs in a minority of patients, and most commonly the remission of histological lesion is observed, but with persistence of the lymphocytic infiltrate [36]. This remission occurs slowly and progressively and is influenced by age, the degree of initial injury, and adherence to GFD [34]. Thus, we believe that histological lesions remission could have been slower because of severe injuries on adult patients (Marsh III B and C) at the diagnosis, despite a period of more than twelve months of treatment.

However, Dickey et al. [24] did not observe any differences in serum folate concentrations when comparing patients in total or partial recovery of intestinal villi and control. Furthermore, Hallert et al. [37] observed folate deficiency in 37 % of celiac patients under treatment with GFD for 10 years and suggest that other factors, such as inadequate nutrient consumption, may have contributed to this results.

In this study, although we did not observe difference in nutrient intake between celiac patients and controls, 100 and 56.6 % of celiac patients showed inadequate folate and vitamin B_12_ consumption, respectively. Despite the importance of the GFD on celiac patient’s health, there is a paucity of studies assessing the nutritional adequacy of this diet.

Recent studies that compared the consumption of vitamins in treated celiac patients and healthy controls observed deficiency on vitamins B_1_, B_2_, B_6_, and folate intake [38–40]. However, to the extent of our knowledge, only two studies [41, 42] compared the vitamin intake adequacy in relation to nutritional recommendations, in order to show the real supply of nutrients by GFD.

The hypothesis for these nutritional deficiencies are based on intake of gluten-free products, which are often produced with refined flours with no fortification [37, 43], and the inadequacy of the habits and food choices of celiac patients [44, 45]. Thompson [46], analyzing the nutritional composition of wheat replacer food concluded that most of these products were not a natural source of or enriched with vitamins B_1_, B_2_, B_3_, folate, and iron. Still, Lee et al. [47] showed that the celiac population did not reach the minimum recommendation of six servings of whole grains a day, needed to achieve adequate intake of folate.

Associated with these factors, a low demand for nutritional counseling with a health-care professional, such as nutritionist, as found in this study, may favor for cases of inadequate food consumption. GFD is simple in its principles; however, to completely eliminate all foods and ingredients that contain gluten is a task that requires a lot of effort and commitment [45]. Health professionals have the role of guiding the patients so that GFD could be healthy, interesting, and practical [48]. These goals are difficult to be achieved for patients who are not professionally oriented because the diet imposed is restrictive, and the changes required are difficult and permanent [33].

The hyperhomocysteinemia occurrence may be related to both vitamin deficiency and genetic abnormalities of methyltetrahydrofolate reductase (MTHFR) and methionine synthase reductase (MTRR) enzymes [49]. Despite of heterozygous and homozygous prevalence of mutations on MTFHR be 42-47 % and 9-17 %, [50] and on MTRR be 50 % and 19-29 %, respectively in the general population, [51] in celiac patients this prevalence is approximately 21 % for both enzymes [52], suggesting that there is no important role of these mutations in developing hyperhomocysteinemia in these patients [28].

Deficiency of folate in the long term may result in the elevation of the serum concentrations of homocysteine with greater risk of developing coronary conditions [53, 54]. Although in our study we observed serum and intake deficiencies of folate in celiac patients, only 1 (6.6 %) patient presented hyperhomocysteinemia. In untreated patients, the prevalence of hyperhomocysteinemia ranged from 20 [28, 54] to 46 % [35]. In treated patients, studies evaluating this prevalence are still scarce. Zanini et al. [35] observed 24 % prevalence of hyperhomocysteinemia in patients under treatment for 1 to 5 years, while Hallert et al. [37] found hyperhomocysteinemia in patients undergoing GFD for 10 years.

Moreover, Dickey et al. [26] and Saibeni et al. [28] observed serum homocysteine reduction after 1 year of GFD, while De Marchi et al. [12] did not observe this effect after 6–8 months of treatment. Thus, more studies are needed to establish the relationship between the adhesion to GFD and serum homocysteine concentrations.

## Conclusion

Our findings demonstrated inadequacy of dietary intake and low-serum levels of folate in celiac patients treated with GFD. It is suggested that more attention should be given to the quality of the nutrients offered by GFD because this constitutes a treatment for life.

## Abbreviations

GFD: Gluten-free diet
BMI: Body mass index
FMI: Fat mass index
DRI: Dietary reference intake
EAR: Estimated average requirement
PLP: Pyridoxal-5-phosphate
